# Prediction of Long-term Cardiac Events by 123I-meta-Iodobenzylguanidine Imaging after acute Myocardial Infarction and Reperfusion Therapy

**DOI:** 10.22038/AOJNMB.2019.33991.1236

**Published:** 2019

**Authors:** Manabu Nakamura, Masahisa Onoguchi, Takayuki Shibutani

**Affiliations:** 1 Department of Medical Examination, Ogaki Municipal Hospital, Ogaki, Japan; 2 Department of Quantum Medical Technology, Graduate School of Medical Sciences, Kanazawa University, Kanazawa, Japan

**Keywords:** Acute myocardial infarction, Long-term cardiac event, Heart-to-mediastinum ratio, Washout rate, ^ 123^I-MIBG imaging

## Abstract

**Objective(s):**

In heart failure, the heart-to-mediastinum (H/M) ratio of the delayed image and washout rate (WR) are well-known as a powerful cardiac event predictors. H/M ratio quantifies the accumulation rate of MIBG in the myocardium and WR quantifies reduction of meta-iodobenzylguanidine (MIBG) accumulation in the heart from the early planar image to the delayed planar images in the ^123^I-MIBG scintigraphy. The present study was conducted to estimate the role of the parameters of cardiac sympathetic imaging by ^123^I-MIBG myocardial scintigraphy in subacute phase of acute myocardial infarction (AMI) in the prediction of cardiac events, particularly in patients who are successfully responded to reperfusion therapy.

**Methods:**

This study was conducted on 145 patients with initial AMI who underwent ^123^I-MIBG myocardial scintigraphy and myocardial single-photon emission computed tomography (SPECT) after successful response to reperfusion therapy. The ^123^I-MIBG myocardial scintigraphy was averagely performed 16±5.8 days after the onset of AMI. The early image was taken 15 min after the intravenous administration of ^123^I-MIBG. Three hours after ^123^I-MIBG administration, an anterior planar delayed SPECT image was obtained. The H/M ratio and WR were calculated based on planar images. In addition, the average WR, defect volume, and extent were calculated from the SPECT image. The end points of the cardiac event was defined as hospitalization due to unstable angina, heart failure progression, myocardial infarction recurrence, malignant arrhythmia and cardiac death.

**Results:**

The follow-up period was 18.4±8.5 months on average, during which 38 (26.2%) cases experienced cardiac events. The results revealed a significant difference between the groups with and without cardiac events in terms of WR and WR (SPECT). Based on the multivariate analysis, WR was the only relevant factor predicting cardiac events. The cumulative event-free rate was significantly lower in the group with the delayed H/M ratio of < 1.74. The cumulative event-free rate were significantly lower in the groups with WR and WR (SPECT) more than 25% and 21.8%, respectively. There was no significant relationship between the cumulative event-free survival rate and the defect size.

**Conclusion:**

In the subacute phase of myocardial infarction, the increased WR of ^123^I-MIBG from the myocardium in planar scintigraphy and SPECT is the predictor of heart failure and cardiac events such as myocardial infarction and recurrence of unstable angina.

## Introduction

Myocardial ischemia and sympathetic nerve activity are known to be closely related. Myocardial ischemia leads to the excessive secretion of norepinephrine (NE) from the sympathetic end of the ischemic part, which promotes myocardial cell damage. The release of NE in myocardial ischemia lasts for several minutes. This incidence is considered as a leak phenomenon due to the breakdown of the uptake-1 mechanism (1) or cell membrane disorder of the sympathetic nerve (2), which reportedly persists from several weeks to several months.

^123^I-metaiodobenzylguanidine (^123^I-MIBG) as a myocardial imaging agent, is a catecholamine analogue and is primarily taken up in the presynaptic vesicles as a tracer via the uptake-1 mechanism, which is the same mechanism applied in the sympathetic nerve and adrenal medulla for NE. Since Wieland et al. (3) reported the accumulation of ^131^I-MIBG in the adrenal medulla, the accumulation of ^131^I or ^123^I-labeled MIBG in the myocardium and decreased MIBG accumulation in the local denervation part have been experimentally conducted (4) and clinically applied. 

In addition, Kline et al. (5) performed the cardiac ^123^I-MIBG imaging on healthy subjects, since ^123^I emits γ rays that are suitable for imaging, the quantitative evaluation of catecholamine activity in the myocardium is facilitated. Merlet et al. (6) proposed a prognostic prediction of chronic heart failure in 1992. they mentioned that heart-to-mediastinum (H/M) uptake ratio obtained from delayed planar MIBG image has prognostic value and is superior to left ventricular ejection fraction (LVEF) values(6-8) and heart rate variability analysis (9, 10). 

The heart-to-mediastinum (H/M) ratio and washout rate (WR), which quantifies the degree of cardiac MIBG accumulation and the rate of reduction from the early anterior planar image to the delayed anterior planar image are used as the parameters of the cardiac sympathetic nerve activity of ^123^I-MIBG. It is well known that the delayed H/M ratio and WR calculated based on the anterior planar images are powerful cardiac event predictors in cases with heart failure, including ischemic heart failure. 

The early H/M ratio reflects the uptake-1 mechanism of catecholamines and myocardium sympathetic activity. 

On the other hand, the delayed H/M ratio, adding the factor of washout, is an index reflecting the distribution and function of cardiac sympathetic system. 

In patients with heart failure, the WR is increased, and the delayed H/M ratio is markedly decreased (11), facilitating the assessment of disease progression as the decline of the left ventricular function, regardless of the underlying disease. However, in cases of advanced heart failure, the evaluation of MIBG myocardial single-photon emission computed tomographic (SPECT) images is difficult due to the poor image quality caused by severely decreased MIBG accumulation, attenuation of inferior wall and the cardiac apex, and also known physiological reduction of MIBG accumulation in the inferior wall (12). However, the clinical significance of subacute phase ^123^I-MIBG myocardial scintigraphy for prognosis evaluation has not yet been clarified in patients with acute myocardial infarction (AMI).

With this background in mind, the present study was conducted to evaluate the parameters of cardiac sympathetic imaging by ^123^I-MIBG myocardial scintigraphy- including MIBG uptake and clearance in patients who are in the initial subacute phase of AMI and successfully responded to reperfusion therapy . It was also examined whether the abnormality could be a predictor of cardiac events after AMI.

## Methods

***Study population***

This study was conducted on 145 patients with initial AMI who underwent ^123^I-MIBG myocardial scintigraphy after successful response to reperfusion therapy. The patients were followed up for prognosis. The study population consisted of 105 males with the mean age of 65±11.3 years. The vessels responsible for infarction included the left anterior descending coronary artery, left coronary artery circumferential branch, and right coronary artery in 59, 24, and 62 cases, respectively. This study was approved by the local Ethics Review Committee (No. 0170223-9).

***MIBG Imaging***

The ^123^I-MIBG myocardial scintigraphy, was performed with a mean of 16±5.8 days after onset of AMI. In the imaging protocol, 111 MBq of each of ^123^I-MIBG and ^201^TlCl was administered intravenously at the time of resting and 15 min later. Subsequently, an early anterior planar image of the chest was acquired, followed by myocardial SPECT imaging. Also, a delayed anterior planar image of the chest was acquired 3 h after administration, followed by SPECT imaging.

The equipment used in the current study was a 2-detector camera system (ADAC Vertex Plus, manufactured by PHILIPS/ADAC) and a low-energy all-purpose collimator was used. The anterior planar image was taken for 5 minutes, with 20% window centered at 159 keV. Simultaneous dual-isotope ^123^I-MIBG/^201^Tl SPECT was performed for SPECT imaging, we set up the energy window of ^123^I-MIBG at 159±10% keV and ^201^Tl at 71±10% keV. 

A total of 32 projection data (in a step and shoot mode, 60 sec/projection, imaging time of 16 min) were obtained with a pixel size of 4.74 mm, a matrix of 128×128, and a zoom of 1.00. ^123^I-MIBG data were reconstructed by the filtered back projection method using a Butterworth filter (order 10, cut off frequency of 0.32 cycles/pixel) as the preprocessing filter and Shepp and Logan filter as the reconstruction filter to create a SPECT image.

***Data analysis***

Parameters for predicting cardiac events were calculated based on the ^123 ^I-MIBG planar and SPECT imaging for the early and delayed images, respectively. From the planar image, early and delayed H/M ratio and WR were calculated by estimating the average count per pixel in the region of interest (ROI) over the left ventricular myocardium and superior mediastinum (6, 13). The WR was calculated by the following equation with the addition of time attenuation correction and background correction: 

WR =((He‒Me)‒(Hd‒Md))/k(He‒Me)×100 (Eq. 1)

*Where*, *He* represents the average heart counts in the early image, *Hd* denotes the average heart counts in the delayed image, *Me* refers to the average count of mediastinum in the early image, *Md* shows the average count of the mediastinum in the delayed image, and *k* delineates time attenuation coefficient (k=0.5 ^t /13^, t: elapsed time

In the SPECT images, the average WR [WR (SPECT)], as well as the defect volume and extent (%) were calculated by dividing the left ventricle into20 segments from the bull’s eye display in the QPS program. The time attenuation correction was performed for WR (SPECT) measurement. The end point of the cardiac event included cardiac death, hospitalization due to unstable angina and heart failure progression, myocardial infarction recurrence, and malignant arrhythmia

***Statistical analysis***

Intergroup comparison the cardiac event and event-free groups for each parameter was performed using the Mann-Whitney U test. These data were also presented as mean and standard deviation. Multivariate analysis was conducted based on the Cox proportional hazards model to verify the cardiac event predictor. Next, based on the receiver operating characteristic (ROC) curve analysis, the optimal cutoff value of each factor related to the occurrence of a cardiac event was determined. 

The optimum cutoff value was considered as the value at which the average values of sensitivity and specificity became the maximum. The cumulative event-free rate grouped by the optimum cutoff value of each factor up and down was examined by the Kaplan-Meier method, and a significant difference test was carried out by logrank test. P-value less than 0.05 was considered statistically significant.

## Results

***Cardiac event group and event-free group***

After the implementation of ^123^I-MIBG myocardial scintigraphy in the subacute phase of AMI, patients were followed up for an average of 18.4±8.5 months (range: 1-32 months). During this period, out of the 145 subjects, 38 (26.2%) cases suffered from cardiac events. The cardiac events observed in the current study included cardiac death (n=6), unstable angina (n=24), exacerbation of heart failure (n=4), recurrence of myocardial infarction (n=3), and malignant arrhythmia (n=1). 

 Patients were divided into two groups based on the presence or absence of cardiac events during the follow up period. The factors were compared based on age, gender difference, responsible blood vessel, follow-up period, and ^123^I-MIBG myocardial scintigraphy. The results revealed a significant difference between the two groups in terms of WR and WR (SPECT) (Table 1).

Results of multivariate analysis based on the Cox proportional hazard model using delayed H/M ratio, WR, WR (SPECT), early defect volume, and early extent, showed that WR was the only cardiac event predictor by ^123^I-MIBG (P=0.04241; Table 2).

***Optimum cutoff value of each factor***

**Figure 1:**
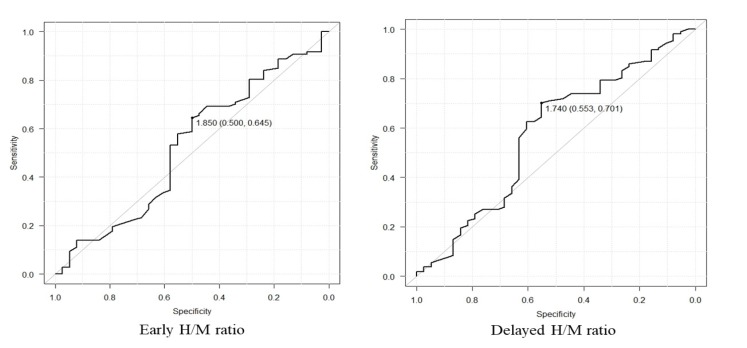
(Left) Receiver operating characteristic(ROC) curve analysis for early heart-to-mediastinum (H/M) ratio showing a low predictive value (AUC=0.524) for cardiac event with an optimal cutoff value of 1.85, yielding a sensitivity of 64.5% and a specificity of 50.0%, (Right) receiver operating characteristic (ROC) curve analysis for delayed heart-to-mediastinum (H/M) ratio showing a low predictive value (AUC=0.576) for cardiac event with an optimal cutoff value of 1.74, yielding a sensitivity of 70.1% and a specificity of 55.3%

**Figure 2:**
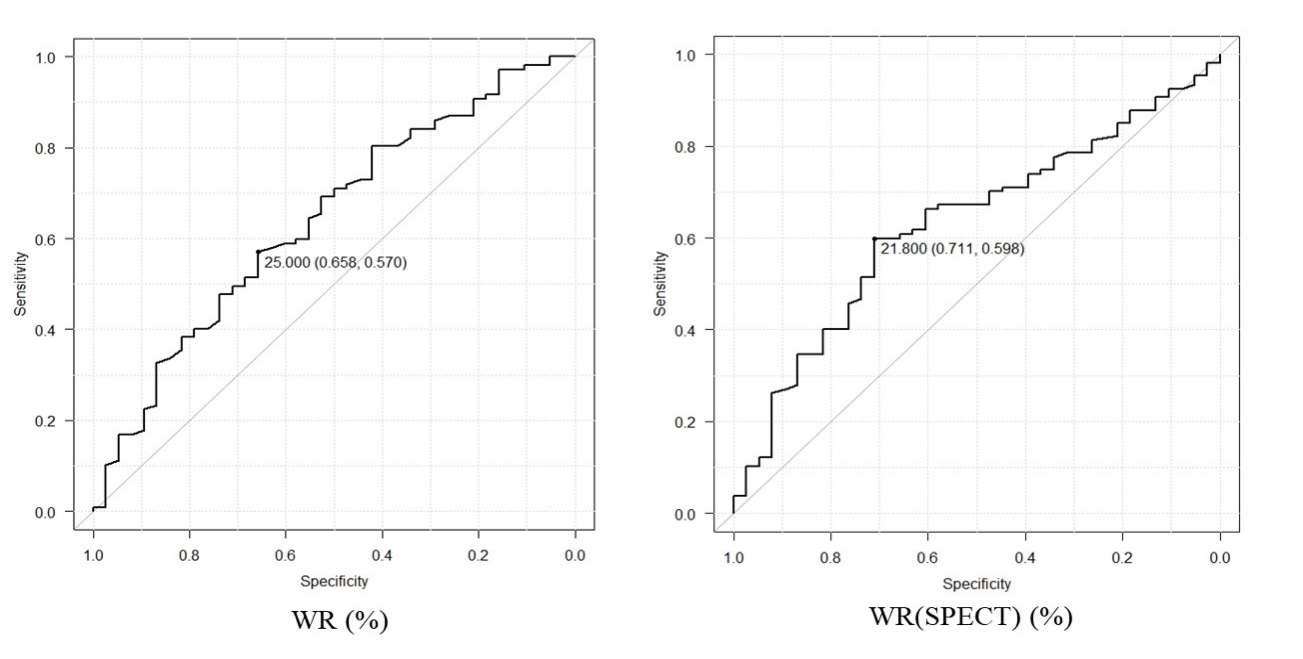
(Left) Receiver operating characteristic (ROC) curve analysis for washout rate showing a slightly low predictive value (AUC=0.64) for cardiac event with an optimal cutoff value of 25.0%, yielding a sensitivity of 57.0% and a specificity of 65.8%, (Right) receiver operating characteristic curve analysis for washout rate (SPECT) showing a slightly low predictive value (AUC=0.63) for cardiac event with an optimal cutoff value of 21.8%, yielding a sensitivity of 59.8% and a specificity of 71.1%

**Figure 3:**
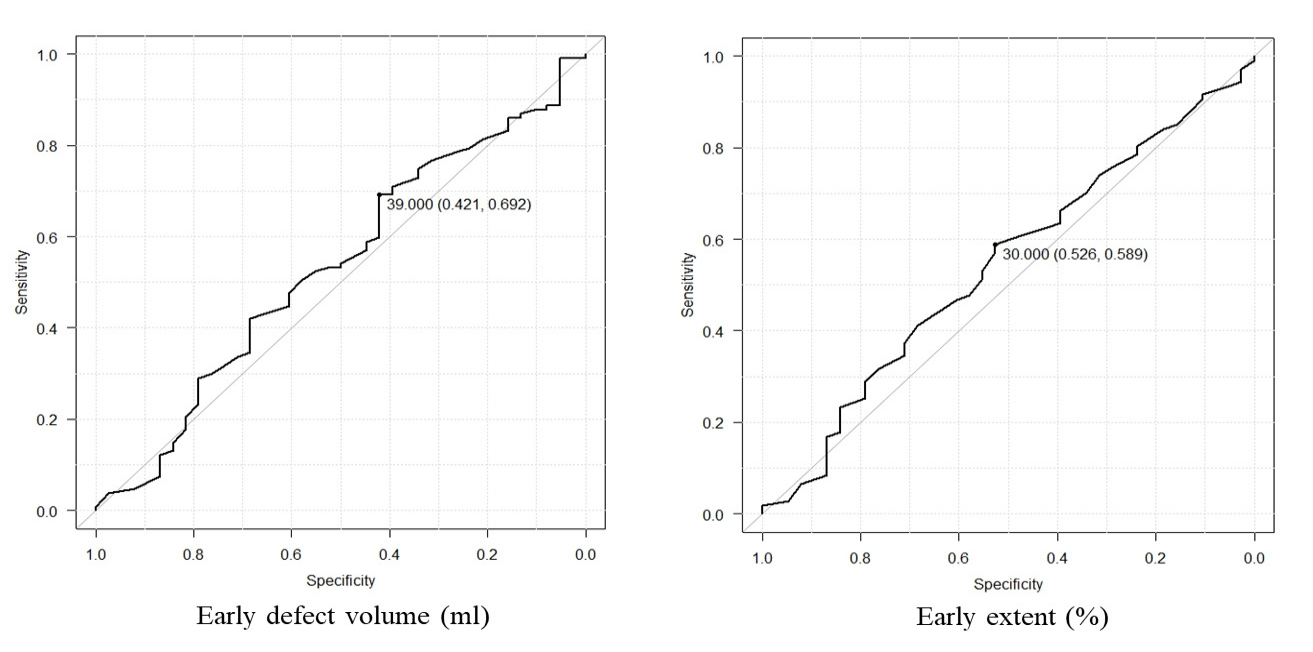
(Left) Receiver operating characteristic (ROC) curve analysis for early defect volume showing a low predictive value (AUC=0.533) for cardiac event with an optimal cutoff value of 39.0 ml, yielding a sensitivity of 69.2% and a specificity of 42.1%, (Right) receiver operating characteristic (ROC) curve analysis for early extent showing a low predictive value (AUC=0.541) for cardiac event with an optimal cutoff value of 30.0%, yielding a sensitivity of 58.9% and a specificity of 52.6%

The optimal cutoff value was determined by ROC curve analysis for early H/M ratio, delayed H/M ratio, WR, WR (SPECT), early defect volume, and early extent, which were 1.85 (AUC=0.524, sensitivity: 64.5%, specificity: 50%) for early H/M ratio, 1.74 (AUC=0.576, sensitivity: 70.1%, specificity: 55.3%; Figure 1) for delayed H/M ratio, 25.0% (AUC=0.64, sensitivity: 57%, specificity: 66%) for WR, 21.8% (AUC=0.63, sensitivity: 60%, specificity: 71%) for WR(SPECT). (Figure 2), 39.0ml (area under the curve 0.533, sensitivity 69%, specificity 42%) for early defect volume and 30.0% (AUC=0.541, sensitivity: 59%, specificity: 53%) for early extent (Figure 3).

***Analysis of cardiac event occurrence***

**Figure 4:**
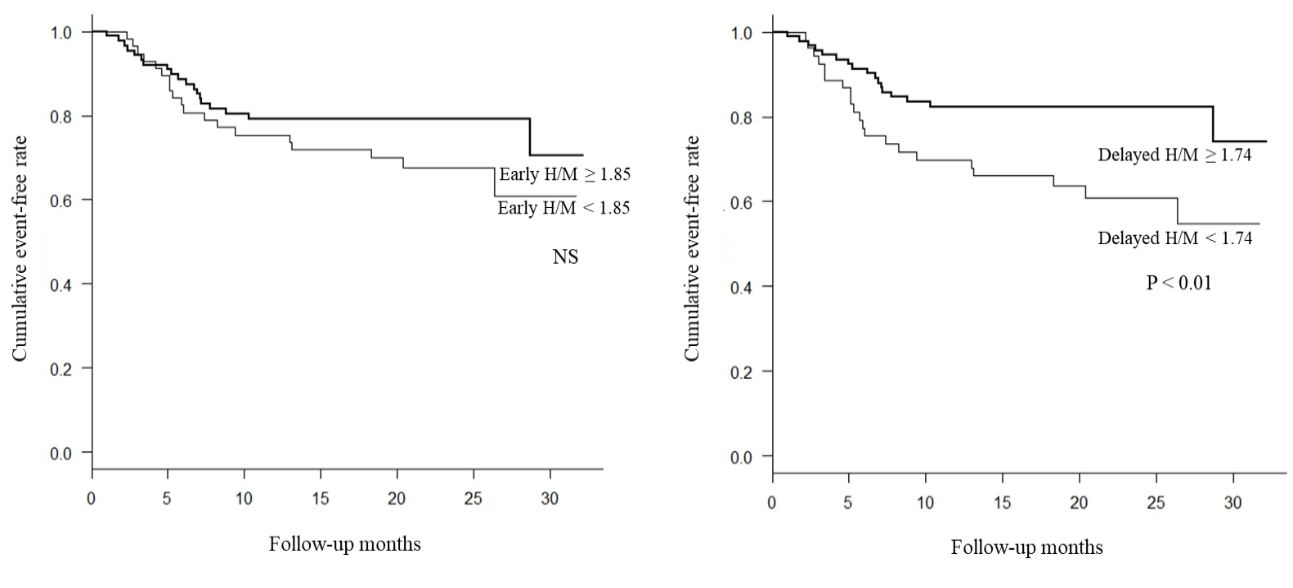
Kaplan-Meier event-free curves for AMI patients stratified into 2 groups. The event-free ratio was significantly lower in patients with a low Delayed H/M ratio (< 1.74) (Right). Early H/M ratio did not have a proper threshold value for significant discrimination of prognosis (Left)

**Figure 5:**
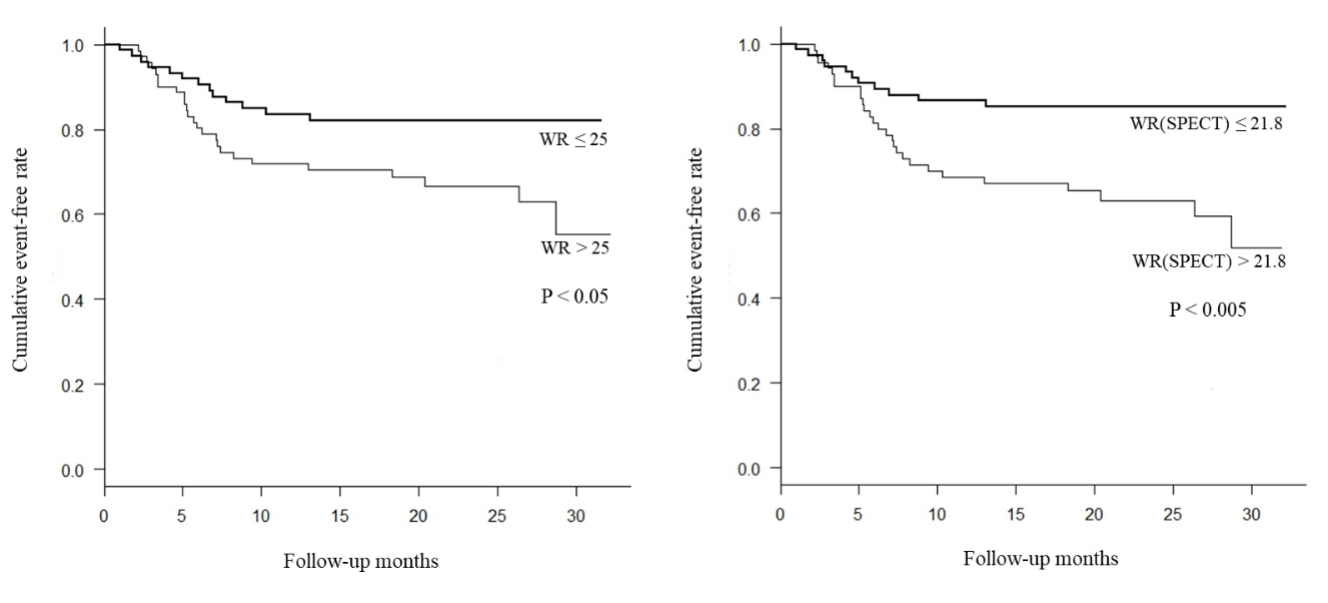
Kaplan-Meier event-free curves for AMI patients stratified into 2 groups. The event-free ratio was significantly lower in patients with a high WR (> 25) and a high WR (SPECT) (> 21.8)

**Figure 6:**
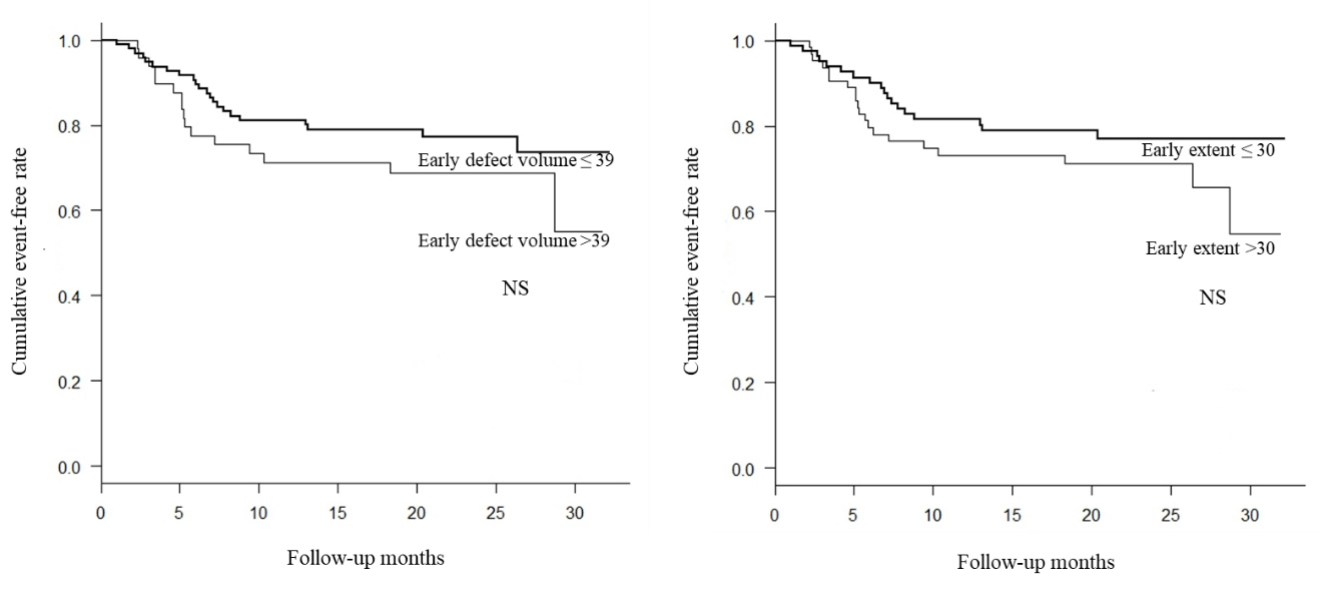
Kaplan-Meier event-free curves for patients with acute myocardial infarction stratified into two groups (Early defect volume and early extent did not have a proper threshold value for discriminating the prognostic value.)

Each factor was divided into two groups based on locating above or below the optimum cutoff value. The cumulative event-free rate was examined with the cardiac event as the end point. As a result, cumulative event-free rate was significantly lower in the group with delayed H/M ratio < 1.74 than in the group with with delayed H/M ratio ≥ 1.74 (Figure 4). The cumulative event-free rate was significantly lower in the group with WR of > 25% than in the group with WR of ≤ 25%, . Again cumulative event-free rate was significantly lower in the group with WR (SPECT) of > 21.8%, compared with WR(SPECT) of ≤ 21.8% (Figure 5). There was no significant correlation between the extent of MIBG defect and total event-free rate (Figure 6).

## Discussion

Factors related to the cardiac sympathetic nerve activity, which may predict the prognosis of AMI were examined in this study. In the current study, 145 first-time AMI patients were followed up for an average of 18.4±8.5 months (with a maximum of 32 months); however, 38 (26.2%) patients experienced cardiac events. The WR and WR (SPECT) were identified as the factors contributing to the prediction of cardiac events. On the other hand, the sites of myocardial infarction, H/M ratio, and MIBG defect size failed to facilitate the prediction of cardiac events. Conventionally, H/M ratio has been used as an index of the myocardial ^123^I-MIBG uptake. Nonetheless, in a study, this index was reported to be inappropriate to be used as a standard value for examining the myocardial uptake. This claim has been supported by a number of reasons, including the alteration of the mediastinal ^123^I-MIBG uptake according to the patients’ age and many other circumstances (14), the wide variation of H/M ratio across institutions (15), and the influence of collimator (16). On the other hand, WR is unlikely to be affected by the differences between the collimators and gamma camera resolution values; therefore, it can be regarded as a more universal index.

**Table 1:** Comparison of Characteristic of Subjects With and Without Cardiac Events

**Characteristic **	**Subjects with Events (n = 38)**	**Subjects without Events (n = 107)**	**P Value**
Age (y)	67±9.8	64±11.8	0.21
Sex (M/F)	25/13	80/27	0.394
Culprit Vessel			
LAD	17 (46%)	42 (39%)	0.69
LCX	8 (21%)	16 (15%)	0.539
RCA	13 (35%)	49 (45%)	0.294
Follow-up months	7.5±6.4	22.3±5.2	< 0.001
Planner Parameters			
Early H/M ratio	1.89±0.26	1.91±0.22	0.66
Delayed H/M ratio	1.78±0.28	1.85±0.24	0.163
WR (%)	29.0±12.5	23.0±11.4	0.010
SPECT Parameters			
WR (SPECT) (%)	24.9±10.3	20.3±13.0	0.017
Early defect volume (ml)	34.6±22.4	32.5±21.8	0.551
Delayed defect volume (ml)	40.2±21.3	37.8±19.2	0.571
Early extent (%)	27.9±14.3	26.0±14.4	0.457
Delayed extent (%)	32.3±13.2	31.5±13.2	0.82

Data are represented as mean ± SD or number

LAD = Left Anterior Descending Artery; LCX = Left Circumflex artery;

RCA = Right Coronary Artery; H/M = Heart-to-Mediastinum; WR =Washout Rate

**Table 2:** Independent predictors of cardiac events in multivariate Cox proportional hazard regression analysis

**Variables**	**Hazard Ratio**	**95% Confidence Interval**	**P Value**
Delayed H/M ratio	1.5010	0.2525 - 8.922	0.65520
WR (%)	1.0510	1.0020 - 1.103	0.04241
WR SPECT (%)	0.9927	0.9598 - 1.027	0.67150
Early defect volume (ml)	0.9929	0.9648 - 1.022	0.62790
Early extent	1.0070	0.9635 - 1.053	0.75140

Abbreviations as in Table 1

Abbreviations as in Table 1

Abbreviations as in Table 1

In the multivariate analysis, WR was identified as the only prognostic factor. Ischemic adenosine-5′-triphosphate (ATP) reduction and leakage phenomenon due to sympathetic cell membrane damage lead to the increased MIBG release from the sympathetic terminal. This results in the impairment of retention function, thereby increasing WR in myocardial ischemia (2).

Based on the optimal threshold, Kaplan-Meier analysis was performed by dividing the patients into two groups of WR greater than 25% and WR lower than 25%, as well as two groups of WR (SPECT) greater than 21.8% and WR (SPECT) lower than 21.8%. The results suggested that the categories with more rates of MIBG washout in both groups had significantly poor prognosis. Furthermore, the increase in WR could be regarded as a predictor of cardiac events.

The physiological meaning of superiority of WR over the other indicators in the prediction of cardiac events after AMI is not clearly explained. There are few reports regarding the prognostic evaluation of MIBG, specifically in ischemic heart diseases. Kobayashi (17) investigated 127 patients with ischemic heart diseases undergoing ^123^I-MIBG myocardial scintigraphy to determine the relationship between prognosis and ^123^I-MIBG index during the mean observational period of 31±18 months. In the mentioned study, death showed a significant correlation with both delayed H/M ratio and WR. 

Using Kaplan-Meier survival analysis, cases with the delayed H/M ratio value of ≤ 1.45, showed significantly poor survival compared with cases with delayed H/M ratio of >1.46 . In addition, patients with a WR of ≥51% had a poor prognosis. However, classification based on delayed H/M ratio resulted in a more effective prognostic prediction than WR-based classification. Imamura et al. (18) examined early H/M ratio, delayed H/M ratio, and WR in MIBG imaging, along with other hemodynamic factors and neurohumoral factors for the prognostic prediction of chronic heart failure. They reported WR as the most useful cardiac event predictor. 

In addition, Momose et al. (19) examined the relationship between early H/M ratio, delayed H/M ratio, WR and the number of segments with myocardial perfusion defect on SPECT image and prognosic value of ^123^I-MIBG myocardial scintigraphic findings in 59 DCM patients in a follow up of 24±13 months. As a result, they reported that WR is more effective as a 123 I-MIBG index for prognosis prediction than using LVEF, delayed H/M, early H/M, and the number of segments with myocardial perfusion defect on SPECT image. It was considered that the degree of sympathetic function in the myocardium has an important meaning in grasping the disease state.

## Conclusion

Based on the findings of the present study, increased WR of ^123^I-MIBG myocardial scintigraphy in both planar and SPECT imaging could not only predict heart failure but also cardiac events, such as myocardial infarction and recurrence of unstable angina, in patients who are in the subacute phase of myocardial infarction.
